# A Manual of the Practice of Medicine

**Published:** 1898-10

**Authors:** 


A MANUAL OF THE PRACTICE OF MEDICINE.—Prepared especially for students by A. A. Stevens, A. M., M. D., Lecturer on Terminology and Instruction in Physical Diagnosis, in the University of Pennsylvania; Demonstration of Pathology in the Woman's Medical College, Philadelphia; Physician to St, Mary's Hospital, etc., etc. Fourth Edition, Revised. Illustrated. Published by W. B. Saunders, Philadelphia. Pages-500. Price, cloth, $2.50.

This work, first published in 1892, has rapidly gained popularity, among those for whom it was designed, viz: medical students. In compactness, succinctness and comprehensiveness, it more resembles the old familiar student's manual. The main facts of medical practice are prominently set forth, and the work embraces many subjects not usually discussed in text books on practice. The revised edition is recommended to medical students without reserve. J.

